# LC–MS/MS-based metabolomic profiling of *Syzygium cumini* (L.) Skeels identifies key phytochemicals associated with α-amylase inhibition and antioxidant activity

**DOI:** 10.1038/s41598-026-55512-x

**Published:** 2026-05-26

**Authors:** Ajay Kumar, Kanhaiya Singh, Jai Prakash, Amit Kumar Goswami, Vishaw Bandhu Patel, Amit Kumar Singh, Yamini Yadav, Aditya Dnyaneshwar Ingole, Bhupendra Sagore

**Affiliations:** 1 The Graduate School, ICAR-Indian Agricultural Research Institute, New Delhi, 110012 India; 2 Division of Fruits and Horticultural Technology, ICAR-Indian Agricultural Research Institute, New Delhi, 110012 India; 3 Horticultural Science Division, ICAR-Krishi Anusandhan Bhawan-II, New Delhi, 110012 India; 4 ICAR-National Bureau of Plant Genetic Resources, New Delhi, 110012 India; 5 Division of Microbiology, ICAR-Indian Agricultural Research Institute, New Delhi, 110012 India; 6 ICAR-Indian Institute of Horticultural Research, Hessaraghatta Lake Post, Bengaluru, Karnataka 560089 India

**Keywords:** LC–MS/MS, Metabolomics, Antioxidant activity, α-amylase inhibition, OPLS-DA, Phenylpropanoids, *Syzygium cumini*, Biochemistry, Biological techniques, Biotechnology, Drug discovery, Plant sciences

## Abstract

Indian blackberry (*Syzygium cumini* (L.) Skeels) possesses immense pharmacological potential. However, understanding the phytochemicals responsible for its biological activities requires detailed characterization of its metabolite composition and associated bioactivities. This study provides a comprehensive LC–MS/MS-based profiling of bioactive compounds and sugars in three contrasting genotypes (PCJ-9, 15, 17) across pulp and seed tissues. We quantified 49 metabolites and identified distinct tissue-specific fingerprints using OPLS-DA (Q^2^ = 0.756–0.988). Seeds were enriched with phenolics (rutin, gallic acid), while pulp dominated in anthocyanins and soluble sugars. Correlation analysis revealed that total flavonoid content (TFC), particularly catechin, along with the phenolic acid protocatechuic acid, was strongly associated with antioxidant and α-amylase inhibitory activities (*r* = 0.95–0.97), compared with total phenolic content. The small-seeded PCJ-15 genotype emerged as the most promising genotype based on its phytochemical composition and in vitro antioxidant and α-amylase inhibitory activities. KEGG pathway analysis revealed a metabolic “hub” in flavonoid biosynthesis and identified a significant inverse correlation between primary sugar accumulation and secondary metabolite synthesis (*r* <  − 0.70). These findings suggest that *S. cumini* may serve as a rich natural source of bioactive phytochemicals with significant antioxidant and α-amylase inhibitory properties. The observed phytochemical and bioactivity profiles highlight its potential for functional food and nutraceutical applications targeting metabolic disorders.

**Supplementary Information:**

The online version contains supplementary material available at 10.1038/s41598-026-55512-x.

## Introduction

Growing interest in plant-derived functional foods and natural therapeutics has intensified research on bioactive-rich fruit species with potential nutraceutical applications^1,2^. *Syzygium cumini* L. (Skeels), widely known as jamun or Indian blackberry, is an underutilized tropical fruit recognized for its medicinal and nutritional value^3^. Traditionally, it has been used in herbal medicine systems to manage cardiometabolic disorders, particularly diabetes and hyperglycemia^4^. Different parts of the plant have been reported to exhibit diverse pharmacological activities, including antihyperglycemic, hypolipidemic, anti-inflammatory, antioxidant, antibacterial, hepatoprotective, antipyretic, and cardioprotective properties^5–7^. These therapeutic potentials are largely attributed to the presence of diverse bioactive compounds, particularly phenolic acids, flavonoids, and anthocyanins^8^.

Recent advances in liquid chromatography coupled with tandem mass spectrometry (LC–MS/MS)-based metabolomics have enabled sensitive and comprehensive profiling of structurally diverse phytochemicals and their functional associations^9,10^. Targeting alpha-amylase inhibition has emerged as a key therapeutic approach to managing postprandial blood glucose spikes^11^. Similarly, natural antioxidants capable of scavenging reactive oxygen species are increasingly being explored as potential alternatives to synthetic therapeutics^12^. In addition, advanced analytical platforms integrating LC–MS/MS with multivariate statistical approaches, including PLS-DA and OPLS-DA, provide improved interpretation of complex metabolomic datasets and facilitate the identification of key discriminatory metabolites associated with biological activities^13,14^.

Although *Syzygium cumini* has been widely recognized for its nutritional and therapeutic potential, previous studies have primarily focused on physicochemical characteristics, antioxidant activity, and targeted phytochemical investigations involving selected metabolite classes such as anthocyanins, phenolics, flavonoids, or carotenoids^15–18^. Nutritional and biochemical evaluations across different jamun landraces and fruit developmental stages have also been reported^18,19^. In addition, LC–MS/MS-based profiling of selected phenolic compounds and sugars has been explored in Myrtaceae fruits, including *S. cumini*, mainly in relation to ripening-associated phytochemical changes and antioxidant properties^20^. However, these investigations largely remained targeted or compound-specific and did not provide an integrated metabolomic framework encompassing simultaneous characterization of phenolic acids, flavonoids, anthocyanins, and sugars.

Furthermore, detailed comparative metabolomic characterization between pulp and seed tissues across contrasting jamun genotypes remains limited. Previous studies also lacked a comprehensive exploration of the metabolic relationship between primary metabolites (sugars) and secondary metabolites, as well as the identification of key discriminating compounds associated with biological activities. Moreover, advanced multivariate and pathway-level analyses, such as OPLS-DA and KEGG pathway interpretation, have rarely been applied in *S. cumini* metabolomic investigations.

Therefore, the present study employed advanced LC–MS/MS-based metabolomic profiling to comprehensively characterize primary and secondary metabolites in pulp and seed tissues of contrasting *S. cumini* genotypes. The study further integrated correlation analysis, OPLS-DA, and KEGG pathway interpretation to identify discriminating metabolites and elucidate metabolic interactions associated with antioxidant and α-amylase inhibitory activities. The novelty of this work lies in its integrated comparative metabolomic framework combining tissue-specific, genotype-specific, and pathway-level analyses with simultaneous profiling of phenolic acids, flavonoids, anthocyanins, and sugars in jamun.

## Result

### Phenolic acids profiling of pulp

**Fig. 1:**
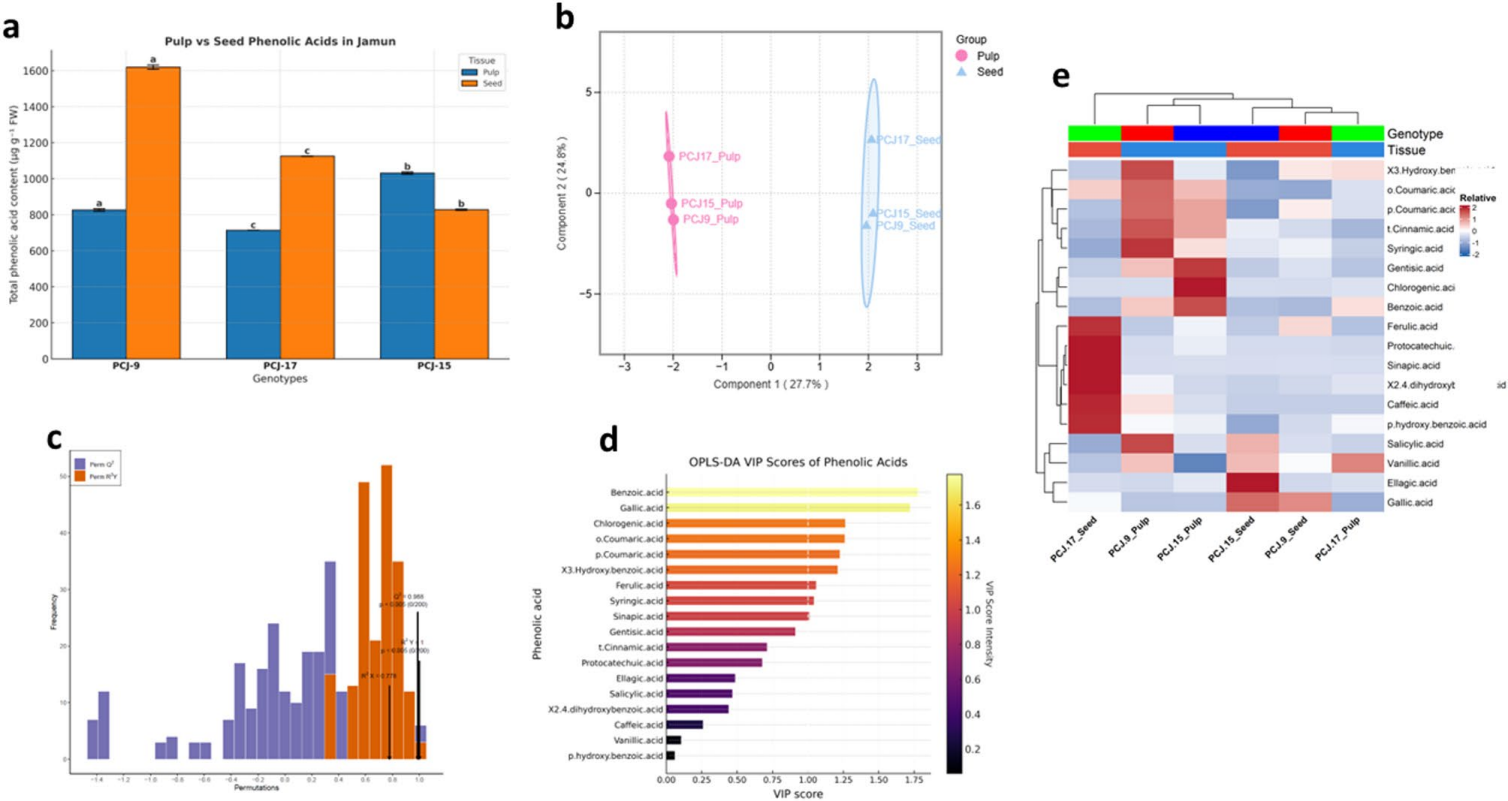
Comparative profiling of phenolic acids in jamun *Syzygium cumini* (L.) Skeels genotypes. (**A**) Total phenolic acid content (µg g^−1^ FW) in pulp and seed tissues of three genotypes (PCJ-9, PCJ-17, and PCJ-15). Bars represent mean ± SD (*n* = 3); different letters indicate significant differences (*p* ≤ 0.05). (**B**) OPLS-DA score plot showing separation between pulp and seed samples. (**C**) OPLS-DA permutation test confirming model robustness (R^2^Y = 1.00; Q^2^ = 0.988; *p* < 0.01). (**D**) OPLS-DA VIP scores highlighting major discriminant phenolic acids. (**E**) Hierarchical heat map depicting relative abundance of individual phenolic acids across genotypes and tissues (red = high, blue = low).

LC–MS/MS profiling of *S. cumini* pulp revealed significant genotype-specific variation in phenolic acid composition (Table 1; Fig. 1). Total phenolic acid content in the pulp ranged from 714.16 µg g^−1^ FW in PCJ-17 to 1030.67 µg g^−1^ FW in PCJ-15, while PCJ-9 exhibited an intermediate level of 831.25 µg g^−1^ FW. Benzoic acid was the predominant phenolic acid in the pulp across all genotypes, with the highest concentration recorded in PCJ-15 (660.14 µg g^−1^ FW), followed by PCJ-9 (483.07 µg g^−1^ FW) and PCJ-17 (451.67 µg g^−1^ FW). Gallic acid was the second most abundant phenolic acid, with concentrations ranging from 223.68 µg g^−1^ FW in PCJ-17 to 313.58 µg g^−1^ FW in PCJ-15. In contrast, vanillic acid content was highest in the pulp of PCJ-17, whereas ellagic and chlorogenic acids were comparatively more abundant in the pulp of PCJ-15.

**Table 1:** Concentration of individual phenolic acids (µg g^−1^ FW) in pulp and seed tissues of three jamun (*Syzygium cumini* (L.) Skeels) genotypes.

Compound	Tissue and genotypes					
	Pulp			Seed		
	PCJ-9	PCJ-17	PCJ-15	PCJ-9	PCJ-17	PCJ-15
Benzoic acid	483.07 ± 6.83^b^	451.67 ± 0.12^c^	660.14 ± 5.60^a^	283.32 ± 11.01^a^	272.48 ± 0.74^c^	280.76 ± 3.31^b^
p hydroxy benzoic acid	0.79 ± 0.02^a^	0.78 ± 0.01^a^	0.75 ± 0.00^b^	0.45 ± 0.01^c^	0.66 ± 0.02^b^	1.46 ± 0.03^a^
Salicylic acid	0.35 ± 0.01^a^	0.21 ± 0.01^b^	0.22 ± 0.00^b^	0.29 ± 0.00^a^	0.22 ± 0.01^b^	0.18 ± 0.00^c^
3 Hydroxy benzoic acid	2.75 ± 0.02^a^	1.77 ± 0.04^b^	1.34 ± 0.01^c^	0.59 ± 0.07^c^	1.70 ± 0.02^a^	1.08 ± 0.05^b^
t Cinnamic acid	8.13 ± 0.10^a^	0.83 ± 0.01^c^	6.00 ± 0.11^b^	2.81 ± 0.05^a^	2.18 ± 0.01^b^	0.92 ± 0.01^c^
2,4 dihydroxy benzoic acid	0.82 ± 0.03^a^	0.65 ± 0.03^b^	0.52 ± 0.05^c^	0.44 ± 0.03^b^	0.52 ± 0.04^b^	2.83 ± 0.08^a^
Gentisic acid	0.65 ± 0.02^b^	0.08 ± 0.01^c^	1.18 ± 0.01^a^	0.12 ± 0.01^b^	0.24 ± 0.01^a^	0.13 ± 0.00^b^
Protocatechuic acid	0.02 ± 0.00^b^	0.01 ± 0.00^c^	0.07 ± 0.00^a^	0.02 ± 0.00^b^	0.02 ± 0.00^b^	0.59 ± 0.02^a^
p Coumaric acid	4.54 ± 0.00^a^	1.64 ± 0.03^c^	3.66 ± 0.02^b^	0.22 ± 0.01^c^	2.54 ± 0.01^a^	1.04 ± 0.01^b^
o Coumaric acid	3.39 ± 0.02^a^	1.01 ± 0.03^c^	2.35 ± 0.02^b^	0.13 ± 0.00^b^	0.08 ± 0.00^c^	2.12 ± 0.01^a^
Vanillic acid	12.41 ± 0.02^b^	14.33 ± 0.05^a^	5.35 ± 0.07^c^	12.63 ± 0.12^a^	10.37 ± 0.01^b^	8.12 ± 0.11^c^
Gallic acid	310.20 ± 1.07^b^	223.68 ± 0.37^c^	313.58 ± 1.92^a^	919.73 ± 4.10^a^	830.05 ± 0.80^b^	492.44 ± 1.03^c^
Caffeic acid	0.15 ± 0.01^a^	0.06 ± 0.01^c^	0.08 ± 0.00^b^	0.06 ± 0.00^b^	0.06 ± 0.00^c^	0.32 ± 0.00^a^
Ferulic acid	0.10 ± 0.00^b^	0.05 ± 0.00^c^	0.33 ± 0.00^a^	0.10 ± 0.00^c^	0.57 ± 0.01^b^	1.26 ± 0.04^a^
Syringic acid	2.62 ± 0.02^a^	0.73 ± 0.03^c^	1.43 ± 0.01^b^	0.96 ± 0.02^b^	1.06 ± 0.02^a^	0.37 ± 0.02^c^
Sinapic acid	0.09 ± 0.00^b^	0.07 ± 0.00^c^	0.13 ± 0.00^a^	0.10 ± 0.00^b^	0.21 ± 0.02^b^	22.67 ± 0.37^a^
Ellagic acid	1.16 ± 0.11^c^	16.54 ± 0.47^b^	31.96 ± 1.33^a^	396.02 ± 3.11^a^	2.44 ± 0.24^c^	10.23 ± 1.14^b^
Chlorogenic acid	0.01 ± 0.00^c^	0.05 ± 0.00^b^	1.58 ± 0.00^a^	0.01 ± 0.00^a^	0.00 ± 0.00^b^	0.00 ± 0.00^b^

*Mean ± SD (*n* = 3) Values with different letters within the same evaluation of same tissue are significantly different (*p* < 0.05).

### Phenolic acids profiling of the seed

Phenolic acids profiling of the seed revealed considerable genotype-specific variation (Table 1; Fig. 1). Total phenolic acid content in the seeds ranged from 826.52 µg g^−1^ FW in PCJ-15 to 1618.00 µg g^−1^ FW in PCJ-9. Gallic acid was the predominant phenolic acid in the seeds, with the highest concentrations recorded in PCJ-9 (919.73 µg g^−1^ FW) and PCJ-17 (830.05 µg g^−1^ FW), whereas PCJ-15 exhibited a comparatively lower concentration (492.44 µg g^−1^ FW). Ellagic acid was most abundant in PCJ-9 (396.02 µg g^−1^ FW) but was detected at minimal levels in PCJ-17. Vanillic acid showed relatively narrow variation among the genotypes, ranging from 8.12 to 12.63 µg g^−1^ FW. In addition, sinapic acid (22.67 µg g^−1^ FW) and protocatechuic acid (0.59 µg g^−1^ FW) were detected exclusively in PCJ-15. Other phenolic acids, including p-coumaric, o-coumaric, cinnamic, syringic, and ferulic acids, exhibited moderate variation across the genotypes, whereas caffeic, salicylic, and chlorogenic acids were detected only in trace amounts.

### Flavonoid profiling of pulp

**Fig. 2:**
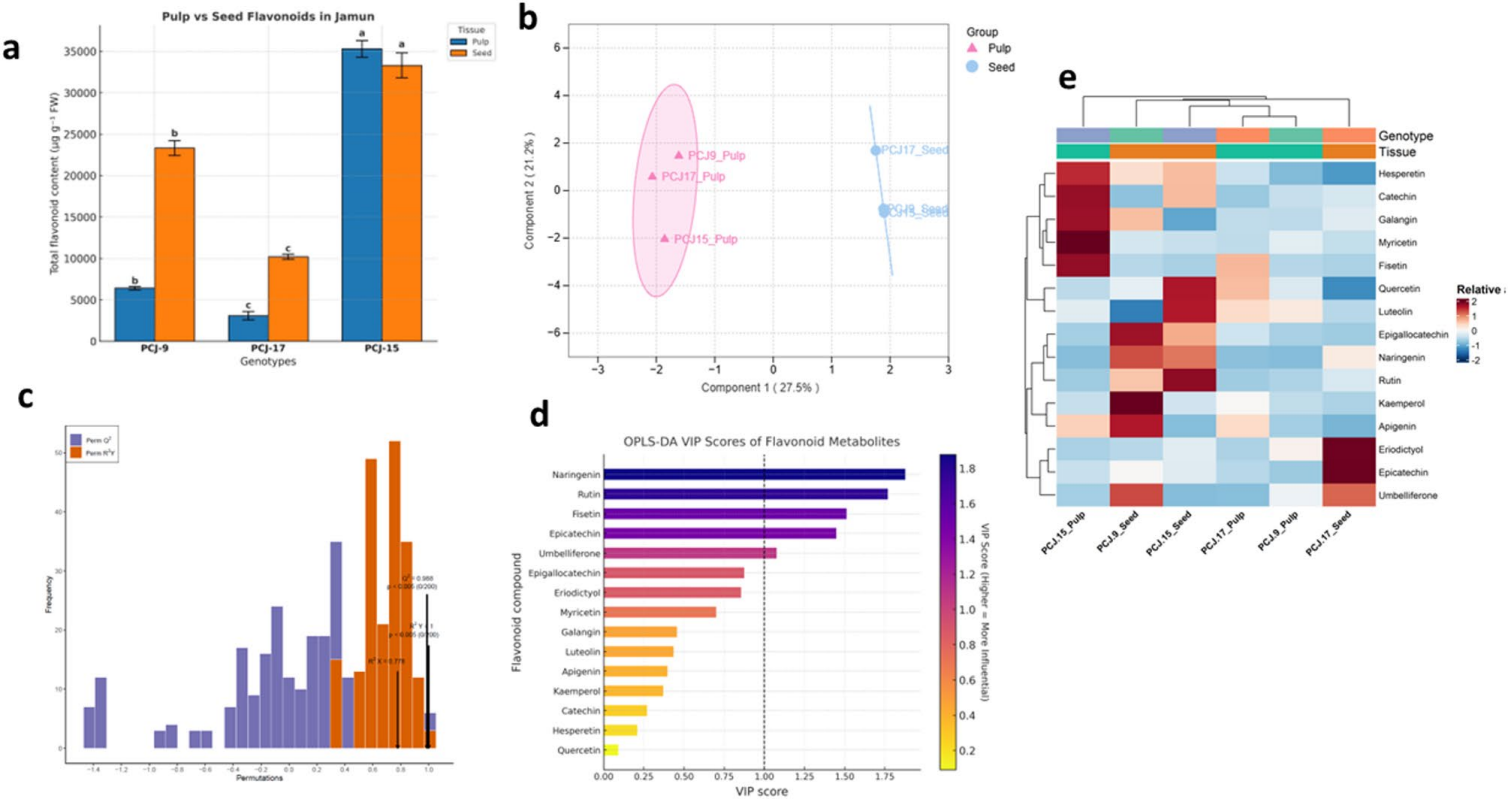
Comparative profiling of flavonoids in jamun *Syzygium cumini* (L.) Skeels genotypes. (**A**) Total flavonoid content (µg g^−1^ FW) in pulp and seed tissues of three genotypes (PCJ-9, PCJ-17, and PCJ-15). Bars represent mean ± SD (*n* = 3); different letters indicate significant differences (*p* ≤ 0.05). (**B**) OPLS-DA score plot showing clear differentiation of pulp and seed samples. (**C**) OPLS-DA permutation test validating model robustness (R^2^Y = 1.00; Q^2^ = 0.988; *p* < 0.01). (**D**) OPLS-DA VIP scores identifying major discriminant flavonoids. (**E**) Heat map clustering illustrating relative abundance of individual flavonoids across tissues (red = high, blue = low).

LC–MS/MS profiling revealed distinct genotype-specific flavonoid profiles in jamun pulp, with 15 flavonoids identified and quantified (Table 2; Fig. 2). Total flavonoid content varied markedly among the genotypes, ranging from 3088.7 ng g^−1^ FW in PCJ-17 to 35,298.3 ng g^−1^ FW in PCJ-15. Among the identified flavonoids, PCJ-15 exhibited exceptionally high concentrations of myricetin (27,111.7 ng g^−1^ FW), catechin (6046.7 ng g^−1^ FW), and hesperetin (696.3 ng g^−1^ FW) compared with the other genotypes. In contrast, PCJ-17 exhibited higher levels of epigallocatechin (766.1 ng g^−1^ FW) along with moderately elevated quercetin content (530.2 ng g^−1^ FW), whereas PCJ-9 showed intermediate concentrations of most flavonoids but comparatively higher rutin content (831.4 ng g^−1^ FW).

**Table 2:** Concentration of individual flavonoids (ng g^−1^ FW) in pulp and seed tissues of three jamun (*Syzygium cumini* (L.) Skeels) genotypes.

Compound	Tissue and genotypes					
	Pulp			Seed		
	PCJ-9	PCJ-17	PCJ-15	PCJ-9	PCJ-17	PCJ-15
Umbelliferone	1.67 ± 0.27^a^	1.28 ± 0.07^b^	1.37 ± 0.15^b^	2.42 ± 0.12^a^	2.34 ± 0.04^a^	1.27 ± 0.13^b^
Apigenin	10.93 ± 1.30^b^	18.41 ± 1.70^a^	19.05 ± 0.62^a^	27.07 ± 3.32^a^	9.20 ± 0.51^b^	9.67 ± 1.09^b^
Galangin	1.44 ± 0.06^b^	1.45 ± 0.09^b^	2.67 ± 0.26^a^	2.06 ± 0.12^a^	1.59 ± 0.16^b^	1.16 ± 0.15^c^
Naringenin	128.59 ± 11.02^b^	169.05 ± 5.71^a^	142.44 ± 4.97^b^	3232.25 ± 97.15^a^	1619.83 ± 11.57^c^	2913.85 ± 77.10^b^
Kaemperol	2.06 ± 0.09^b^	4.73 ± 0.33^a^	2.27 ± 0.19^b^	14.99 ± 0.24^a^	1.44 ± 0.18^c^	2.59 ± 0.09^b^
Luteolin	170.91 ± 11.28	178.45 ± 7.14	151.85 ± 16.27	101.54 ± 14.07^c^	137.94 ± 9.81^b^	232.77 ± 15.75^a^
Fisetin	0.17 ± 0.02^c^	1.44 ± 0.11^b^	2.60 ± 0.09^a^	0.18 ± 0.01^a^	0.10 ± 0.01^b^	0.08 ± 0.00^c^
Eriodictyol	0.72 ± 0.02^a^	0.23 ± 0.02^b^	0.22 ± 0.02^b^	0.28 ± 0.02^c^	2.00 ± 0.12^a^	0.50 ± 0.02^b^
Catechin	870.00 ± 17.37^b^	217.98 ± 9.12^c^	6046.70 ± 203.76^a^	69.46 ± 6.44^c^	1164.72 ± 43.96^b^	3421.79 ± 296.15^a^
Epicatechin	237.88 ± 11.86^c^	416.88 ± 28.47^b^	523.54 ± 26.74^a^	1014.21 ± 38.15^b^	3029.87 ± 24.80^a^	733.39 ± 10.08^c^
Hesperetin	112.72 ± 3.30^c^	219.45 ± 14.30^b^	696.33 ± 20.96^a^	407.93 ± 40.41^b^	46.71 ± 2.18^c^	480.50 ± 15.04^a^
Quercetin	380.16 ± 25.20^b^	530.20 ± 13.74^a^	341.71 ± 20.89^b^	401.13 ± 8.54^b^	221.78 ± 6.88^c^	695.51 ± 10.86^a^
Epigallocatechin	213.72 ± 14.03^b^	766.12 ± 26.29^a^	157.09 ± 12.02^c^	5720.11 ± 113.39^a^	91.98 ± 8.23^c^	3479.17 ± 164.23^b^
Myricetin	3454.74 ± 327.23^b^	384.10 ± 22.78^c^	27,111.70 ± 48.53^a^	1959.91 ± 64.10^a^	637.93 ± 9.68^c^	1002.67 ± 22.42^b^
Rutin	831.38 ± 14.95^a^	178.93 ± 4.61^b^	98.75 ± 6.35^c^	10,369.12 ± 40.25^b^	3230.42 ± 92.51^c^	20,331.19 ± 364.36^a^

*Mean ± SD (*n* = 3) Values with different letters within the same evaluation of same tissue are significantly different (*p* < 0.05).

### Flavonoid profiling of the seed

Seed flavonoid composition exhibited pronounced genotype-specific variation among the evaluated *Syzygium cumini* genotypes (Table 2; Fig. 2). The highest total flavonoid content was recorded in PCJ-15 (33,306.11 ng g^−1^ FW), followed by PCJ-9 (23,322.66 ng g^−1^ FW) and PCJ-17 (10,197.85 ng g^−1^ FW). Rutin was identified as the predominant flavonoid in seed tissues, with maximum content 20,331.2 ng g^−1^ FW in PCJ-15, compared with 10,369.1 ng g^−1^ FW in PCJ-9 and 3,230.4 ng g^−1^ FW in PCJ-17. Moreover, PCJ-9 was characterized by comparatively higher levels of naringenin (3,232.3 ng g^−1^ FW) and epigallocatechin (5,720.1 ng g^−1^ FW), whereas PCJ-17 accumulated higher levels of epicatechin (3,029.9 ng g^−1^ FW). In contrast, PCJ-15 contained the highest concentrations of catechin, quercetin, and hesperetin among the studied genotypes. Several minor flavonoids also demonstrated distinct genotype-specific distribution patterns, including apigenin in PCJ-9, luteolin in PCJ-15, and eriodictyol in PCJ-17.

### Anthocyanin profiling of pulp

**Fig. 3:**
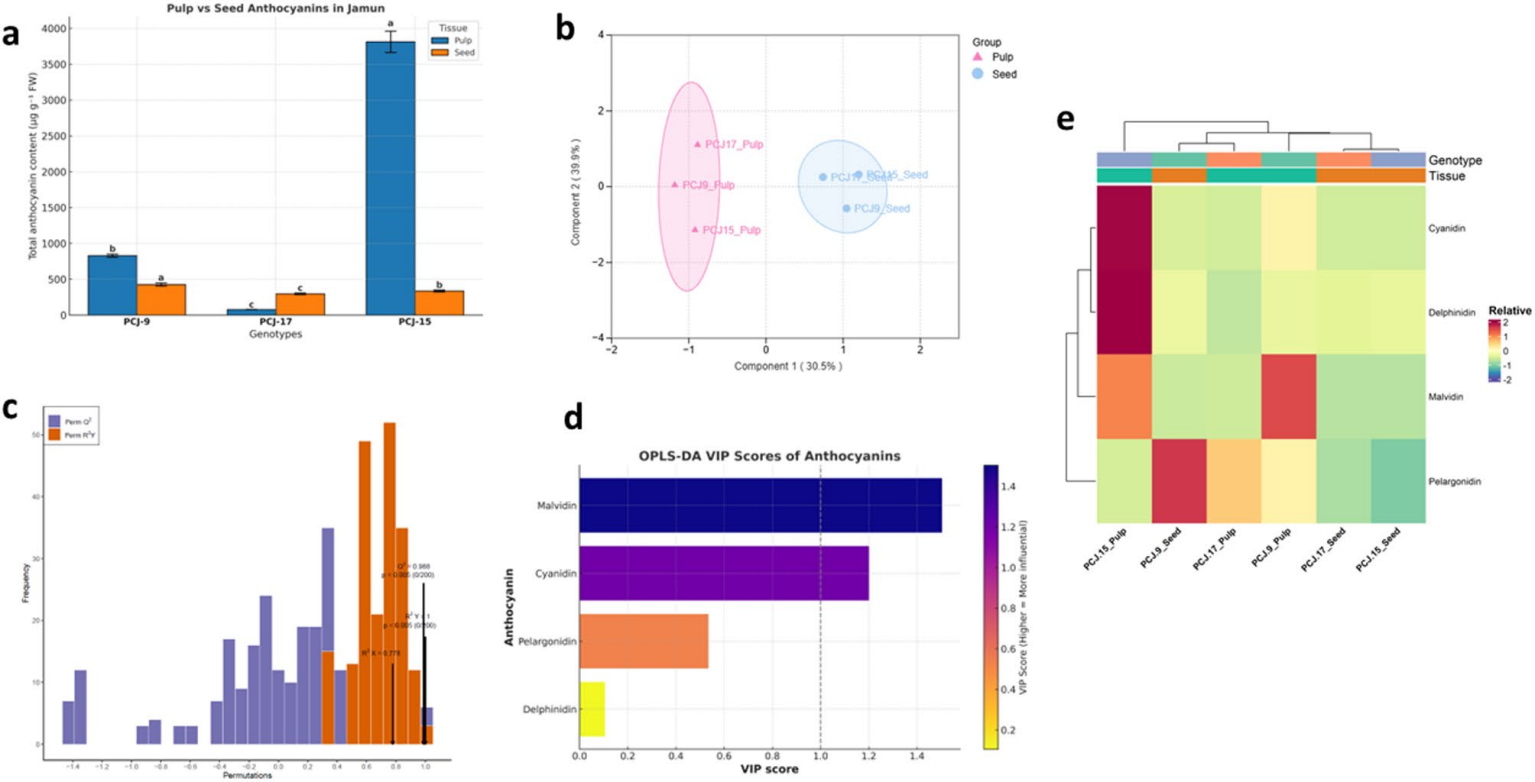
Comparative profiling of anthocyanins in jamun *Syzygium cumini* (L.) Skeels genotypes. (**A**) Total anthocyanin content (µg g^−1^ FW) in pulp and seed tissues of three genotypes (PCJ-9, PCJ-17, and PCJ-15). Bars represent mean ± SD (*n* = 3); different letters indicate significant differences (*p* ≤ 0.05). (**B**) OPLS-DA score plot showing distinct separation between pulp and seed samples based on anthocyanin profiles. (**C**) OPLS-DA permutation test validating model robustness (R^2^Y = 1.00; Q^2^ = 0.988; *p* < 0.01). (**D**) OPLS-DA VIP scores highlighting key discriminant anthocyanins contributing to class separation. (**E**) Heat-map clustering illustrating relative abundance of individual anthocyanins across genotypes and tissues (red = high, blue = low).

The pulp anthocyanin profile showed clear genotypic variation among the studied *Syzygium cumini* genotypes (Table 3; Fig. 3). PCJ-15 exhibited the highest total anthocyanin content (3812.77 µg g^−1^ FW), primarily due to elevated concentrations of cyanidin (1076.03 µg g^−1^ FW) and delphinidin (2601.55 µg g^−1^ FW). In comparison, PCJ-9 showed intermediate concentrations of cyanidin (296.85 µg g^−1^ FW) and delphinidin (363.39 µg g^−1^ FW), resulting in a total anthocyanin content of 829.36 µg g^−1^ FW. Conversely, PCJ-17 contained substantially lower concentrations of cyanidin (6.27 µg g^−1^ FW) and delphinidin (58.31 µg g^−1^ FW), with a total anthocyanin content of only 76.92 µg g^−1^ FW. Malvidin concentration was highest in PCJ-9 (168.86 µg g^−1^ FW), whereas pelargonidin exhibited minimal variation among the genotypes, ranging from 0.16 µg g^−1^ FW in PCJ-15 to 0.32 µg g^−1^ FW in PCJ-17**.**

**Table 3:** Concentration of individual anthocyanins (µg g^−1^ FW) in pulp and seed tissues of three jamun (*Syzygium cumini* (L.) Skeels) genotypes.

Compound	Tissue and genotypes					
	Pulp			Seed		
	PCJ-9	PCJ-17	PCJ-15	PCJ-9	PCJ-17	PCJ-15
Pelargonidin	0.26 ± 0.03^b^	0.32 ± 0.02^a^	0.16 ± 0.02^c^	0.48 ± 0.02^a^	0.12 ± 0.02^b^	0.08 ± 0.01^c^
Cyanidin	296.85 ± 7.34^b^	6.27 ± 0.37^c^	1076.03 ± 26.30^a^	28.36 ± 1.75^a^	3.30 ± 0.33^b^	2.95 ± 0.29^b^
Delphinidin	363.39 ± 12.49^b^	58.31 ± 0.90^c^	2601.55 ± 119.29^a^	387.10 ± 19.76^a^	292.52 ± 10.57^c^	333.43 ± 12.28^b^
Malvidin	168.86 ± 1.25^a^	12.02 ± 0.33^c^	135.03 ± 3.03^b^	10.43 ± 0.15^a^	0.55 ± 0.04^b^	0.24 ± 0.08^c^

*Mean ± SD (*n* = 3) Values with different letters within the same evaluation of same tissue are significantly different (*p* < 0.05).

### Anthocyanin profiling of the seed

Distinct differences in seed anthocyanin composition were observed across the *Syzygium cumini* genotypes (Table 3; Fig. 3). Delphinidin was identified as the predominant anthocyanin in seed tissues, with the highest concentration recorded in PCJ-9 (387.10 µg g^−1^ FW), followed by PCJ-15 (333.43 µg g^−1^ FW) and PCJ-17 (292.52 µg g^−1^ FW). Cyanidin was detected at comparatively lower concentrations across all genotypes, although its maximum concentration was also observed in PCJ-9 (28.36 µg g^−1^ FW). Pelargonidin and malvidin were present as minor anthocyanins but consistently exhibited relatively higher levels in PCJ-9 than in the other genotypes. Total seed anthocyanin content was highest in PCJ-9 (426.37 µg g^−1^ FW), followed by PCJ-15 (336.70 µg g^−1^ FW) and PCJ-17 (296.49 µg g^−1^ FW).

### Sugar profiling of pulp

**Fig. 4:**
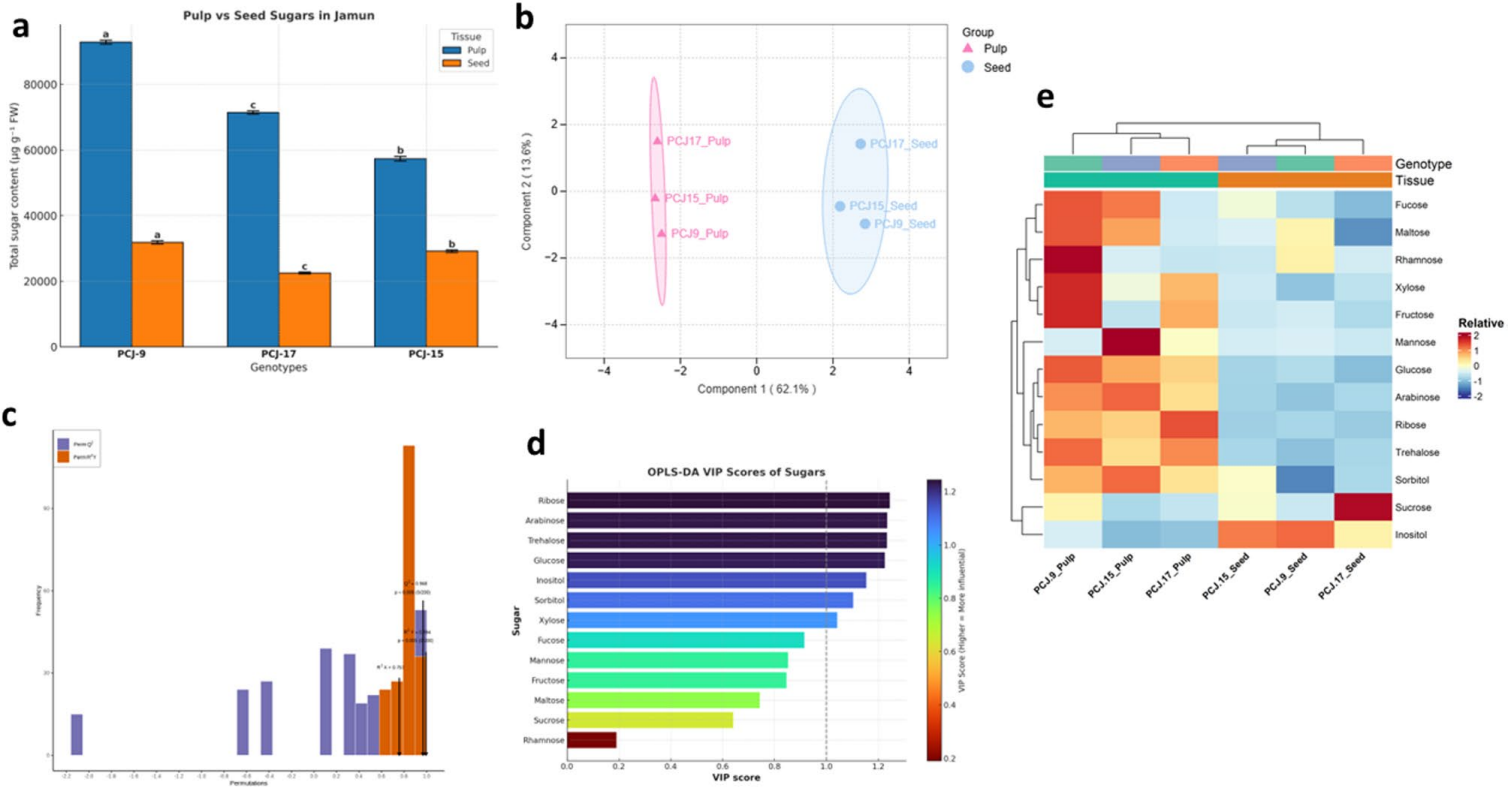
Comparative profiling of sugars in jamun *Syzygium cumini* (L.) Skeels genotypes. (**A**) Total sugar content (µg g^−1^ FW) in pulp and seed tissues of three genotypes (PCJ-9, PCJ-17, and PCJ-15). Bars represent mean ± SD (*n* = 3); different letters indicate significant differences (*p* ≤ 0.05). (**B**) OPLS-DA score plot showing clear separation of pulp and seed samples based on sugar composition. (**C**) OPLS-DA permutation test validating model reliability (R^2^Y = 1.00; Q^2^ = 0.988; *p* < 0.01). (**D**) OPLS-DA VIP scores identifying key discriminant sugars contributing to group differentiation. (**E**) Heat-map clustering showing relative abundance of individual sugars across genotypes and tissues (red = high, blue = low).

Pulp sugar profiles varied substantially across the *Syzygium cumini* genotypes (Table 4; Fig. 4). Among the monosaccharides, ribose sugar ranged from 41.22 µg g^−1^ FW in PCJ-15 to 62.55 µg g^−1^ FW in PCJ-17, while PCJ-9 exhibited an intermediate concentration of 46.26 µg g^−1^ FW. Arabinose and xylose were comparatively lower concentrations across all genotypes. The highest arabinose level (3.93 µg g^−1^ FW) was recorded in PCJ-15, whereas xylose concentration was lowest in the same genotype (0.83 µg g^−1^ FW). Rhamnose and fucose also varied substantially among the genotypes, with PCJ-9 exhibiting the highest concentrations of both sugars at 23.57 and 10.85 µg g^−1^ FW, respectively.

**Table 4:** Concentration of individual sugars (µg g^−1^ FW) in pulp and seed tissues of three jamun (*Syzygium cumini* (L.) Skeels) genotypes.

Compound	Tissue and genotypes					
	Pulp			Seed		
	PCJ-9	PCJ-17	PCJ-15	PCJ-9	PCJ-17	PCJ-15
Ribose	46.26 ± 1.08^b^	62.55 ± 0.52^a^	41.22 ± 0.70^c^	8.06 ± 0.02^a^	5.54 ± 0.08^c^	6.45 ± 0.04^b^
Arabinose	3.69 ± 0.24^a^	3.16 ± 0.34^b^	3.93 ± 0.19^a^	1.91 ± 0.09	2.07 ± 0.11	2.03 ± 0.07
Xylose	1.24 ± 0.08^a^	1.03 ± 0.02^b^	0.83 ± 0.09^c^	0.66 ± 0.01^b^	0.73 ± 0.04^a^	0.76 ± 0.04^a^
Rhamnose	23.57 ± 0.40^a^	2.35 ± 0.10^c^	3.47 ± 0.17^b^	8.84 ± 0.06^a^	3.26 ± 0.11^b^	2.58 ± 0.03^c^
Fucose	10.85 ± 0.14^a^	5.31 ± 0.27^c^	10.18 ± 0.17^b^	4.86 ± 0.07^b^	3.75 ± 0.23^c^	6.35 ± 0.07^a^
Fructose	73,960.67 ± 283.14^a^	53,278.19 ± 238.07^b^	19,567.64 ± 201.02^c^	22,841.97 ± 475.34^a^	16,240.59 ± 136.85^c^	20,948.60 ± 75.73^b^
Glucose	15,465.50 ± 200.26^a^	11,312.27 ± 44.59^c^	12,893.41 ± 30.38^b^	4958.21 ± 80.78^a^	3454.92 ± 40.26^c^	4458.50 ± 8.96^b^
Mannose	2690.56 ± 54.93^c^	6326.92 ± 103.21^b^	24,372.95 ± 118.24^a^	2818.02 ± 7.83^a^	1886.95 ± 13.47^c^	2517.64 ± 57.72^b^
Inositol	423.88 ± 8.31^a^	242.02 ± 3.66^b^	213.52 ± 1.22^c^	1028.04 ± 25.32^a^	648.51 ± 12.44^c^	982.45 ± 19.94^b^
Sorbitol	208.35 ± 4.07^b^	195.19 ± 1.19^c^	224.17 ± 1.48^a^	133.85 ± 3.94^c^	156.92 ± 1.95^b^	181.23 ± 0.88^a^
Sucrose	45.80 ± 0.55^a^	15.91 ± 0.21^b^	8.94 ± 0.05^c^	17.97 ± 0.08^c^	117.53 ± 1.34^a^	37.51 ± 0.63^b^
Maltose	26.25 ± 0.43^a^	11.61 ± 0.03^c^	22.42 ± 0.11^b^	16.69 ± 0.13^a^	4.67 ± 0.07^c^	12.17 ± 0.14^b^

*Mean ± SD (*n* = 3) Values with different letters within the same evaluation of same tissue are significantly different (*p* < 0.05).

Fructose was the predominant reducing sugar in the pulp and exhibited pronounced variation among the genotypes, ranging from 19,567.64 µg g^−1^ FW in PCJ-15 to 73,960.67 µg g^−1^ FW in PCJ-9. Moreover, PCJ-17 showed an intermediate fructose level (53,278.19 µg g^−1^ FW). Glucose and mannose also varied markedly across the genotypes, with the highest glucose content recorded in PCJ-9 (15,465.50 µg g^−1^ FW), whereas PCJ-15 exhibited the highest mannose level (24,372.95 µg g^−1^ FW).

Sugar alcohols, including inositol and sorbitol, were detected at relatively low levels across all genotypes. The highest inositol content was observed in PCJ-9, whereas PCJ-15 exhibited the highest sorbitol level. Among the disaccharides, sucrose content was highest in PCJ-9 (45.80 µg g^−1^ FW) and lowest in PCJ-15 (8.94 µg g^−1^ FW). Overall, total sugar content in the pulp varied substantially among the genotypes, with the highest level recorded in PCJ-9 (92,906.81 µg g^−1^ FW), followed by PCJ-17 (71,456.69 µg g^−1^ FW) and PCJ-15 (57,362.83 µg g^−1^ FW).

### Sugars profiling of the seed

The seed sugar profile revealed considerable diversity across the *Syzygium cumini* genotypes (Table 4; Fig. 4). Among the monosaccharides, ribose content ranged from 5.54 µg g^−1^ FW in PCJ-17 to 8.06 µg g^−1^ FW in PCJ-9. Arabinose and xylose were detected at comparatively lower levels across all genotypes, with the highest xylose content recorded in PCJ-15. Rhamnose content was highest in PCJ-9 (8.84 µg g^−1^ FW) and lowest in PCJ-15 (2.58 µg g^−1^ FW), whereas fucose content varied from 3.75 µg g^−1^ FW in PCJ-17 to 6.35 µg g^−1^ FW in PCJ-15.

Among the reducing sugars, fructose predominated in seeds and was highest in PCJ-9 (22,841.97 µg/g FW), followed by PCJ-15 (20,948.60 µg/g FW) and PCJ-17 (16,240.59 µg/g FW). Glucose and mannose were also highest in PCJ-9 at 4,958.21 µg/g FW and 2,818.02 µg/g FW, respectively. Sugar alcohols were present in trace quantities. The disaccharide sugar, sucrose, was highest in PCJ-17 (117.53 µg/g FW) and lowest in PCJ-9 (17.97 µg/g FW). The maximum difference was found in PCJ-9 (92,906.81 µg/g FW in pulp vs 31,839.16 µg/g FW in seed).

### OPLS-DA analysis of metabolite profiles

#### OPLS-DA score plots revealing tissue-specific metabolite separation

The OPLS-DA score plots reflecting clear separation of metabolites between pulp and seed samples. Component 1 represented the major variation that explained the discrimination, while Component 2 represented orthogonal variation unrelated to class separation. For phenolic acids, Component 1 explained 27.7% of the total variation. In comparison, Component 2 explained 24.6% (Fig. 1d). In flavonoids, Component 1 contributed to 27.5%, while Component 2 captured 21.2% of the orthogonal variance (Fig. 2d). For anthocyanins, Component 1 explained 30.5%. In comparison, Component 2 explained 39.9% (Fig. 3d). In sugar metabolites, Component 1 contributed a comparatively larger proportion, 62.1%. In comparison, Component 2 accounted for 13.6% (Fig. 4d).

#### OPLS-DA model validation and predictive performance

OPLS-DA models for phenolic acids, flavonoids, anthocyanins, and sugars produced high R^2^ and Q^2^ values with significant class discrimination. Phenolic acids, the model gave R^2^X = 0.783, R^2^Y = 0.999, Q^2^ = 0.756 (Fig. 1c). For flavonoids, the values were R^2^X = 0.778, R^2^Y = 1.000 and Q^2^ = 0.988 (Fig. 2c), and for anthocyanins, the values were R^2^X = 0.778, R^2^Y = 1.000, Q^2^ = 0.988 (Fig. 3c). The model for sugars gave R^2^X = 0.757, R^2^Y = 0.984 and Q^2^ = 0.968 (Fig. 4c). Permutation testing confirmed the statistical significance of all models (*p* < 0.005, 0/200 permutations).

#### VIP score analysis for identification of key discriminant metabolites

The OPLS-DA-based VIP score analysis identified the key metabolites contributing to sample discrimination between pulp and seed. In the case of phenolic acids, benzoic (VIP = 1.78), gallic (1.72), chlorogenic (1.26), o-coumaric (1.26), p-coumaric (1.22), sinapic (1.01), and syringic acid (1.04) were the most influential contributors (Fig. 1d). For flavonoids, naringenin (VIP = 1.88), rutin (1.77), fisetin (1.51), epicatechin (1.45), and umbelliferone (1.08) were the major discriminating metabolites. Whereas epigallocatechin, eriodictyol, and myricetin showed moderate VIP score (0.70–0.87) (Fig. 2d). In the case of anthocyanins, malvidin (VIP = 1.50) and cyanidin (VIP = 1.20) were the major discriminants (Fig. 3d). For sugars, arabinose, glucose, inositol, ribose, sorbitol, trehalose, and xylose (VIP > 1) were the most important variables, while fructose, fucose, mannose, maltose, sucrose, and rhamnose contributed minimally (VIP < 1) (Fig. 4d).

#### Antioxidant and α-amylase inhibition activities

The antioxidant (DPPH) and α-amylase inhibition activities of pulp and seed extracts from three jamun genotypes are presented in Table 5. Significant differences (*p* < 0.05) were observed across genotypes and tissues.

**Table 5:** Antioxidant (DPPH), α-amylase inhibition activities, and IC_50_ values of pulp and seed extracts from three jamun (*Syzygium cumini* (L.) Skeels) genotypes.

Genotype	Tissue	DPPH scavenging (%)	Inhibition (%)	IC50
PCJ-9	Pulp	33.93 ± 0.75^e^	56.52 ± 0.73^e^	133.85 ± 4.83^b^
PCJ-9	Seed	54.14 ± 0.69^c^	67.66 ± 1.23^c^	69.03 ± 0.75^d^
PCJ-15	Pulp	92.84 ± 1.36^b^	85.56 ± 0.97^b^	40.53 ± 1.17^e^
PCJ-15	Seed	96.82 ± 0.38^a^	96.85 ± 1.00^a^	38.57 ± 0.65^e^
PCJ-17	Pulp	20.17 ± 0.63f.	40.55 ± 0.74f.	302.20 ± 3.00^a^
PCJ-17	Seed	41.52 ± 0.77^d^	58.61 ± 0.97^d^	116.69 ± 3.26^c^
SE(d)		0.67	0.78	2.24
CV (%)		1.44	1.41	2.35

Values represent mean ± SD (*n* = 3). Different superscript letters within a column indicate statistically significant differences according to Tukey’s HSD test at *p* < 0.05.

#### DPPH radical scavenging activity

The strongest antioxidant activity was recorded in PCJ-15 seed (96.82 ± 0.38%), followed by PCJ-15 pulp (92.84 ± 1.36%). Moderate activity was observed in PCJ-9 seed (54.14 ± 0.69%), while the lowest values occurred in the pulp samples of PCJ-17 (20.17 ± 0.63%). The most potent antioxidant extracts were PCJ-15 seed (38.57 ± 0.65 µg/mL) and PCJ-15 pulp (40.53 ± 1.17 µg/mL), both of which were significantly lower than the other samples (Table 5).

#### α-amylase inhibition activity

A parallel pattern was observed for α-amylase inhibition. The highest inhibitory activity was found in PCJ-15 seed (96.85 ± 1.00%), followed by PCJ-15 pulp (85.56 ± 0.97%). Moderate inhibition occurred in PCJ-9 seed (67.66 ± 1.23%), whereas the lowest values were recorded in PCJ-17 pulp (40.55 ± 0.74%). The lowest IC_50_ values were observed in PCJ-15 seed (38.57 ± 0.65 µg/mL) and PCJ-15 pulp (40.53 ± 1.17 µg/mL). Higher IC_50_ values were recorded in PCJ-9 seed (69.03 ± 0.75 µg/mL) and PCJ-17 seed (116.69 ± 3.26 µg/mL) (Table 5).

#### Correlation between phytochemical composition and biological activities

**Fig. 5:**
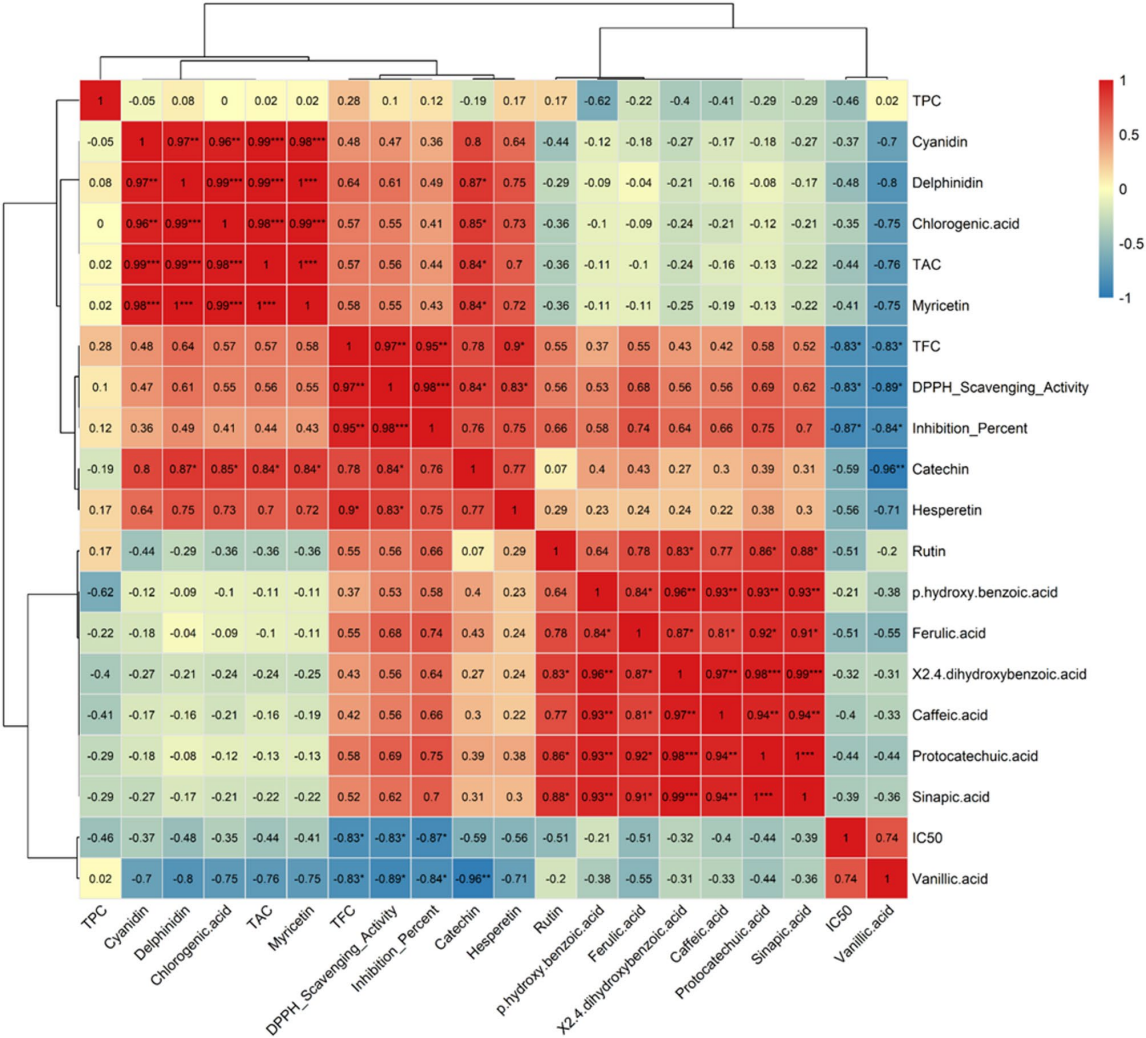
Heatmap of Pearson correlation coefficients (r) among phytochemical constituents, antioxidant activity (DPPH radical scavenging), α-amylase inhibitory activity, and IC_50_ values of different pulp and seed extracts of *Syzygium cumini* evaluated from three biological replicates (*n* = 3). The color gradient represents the magnitude and direction of correlation coefficients, where red indicates positive correlations and blue indicates negative correlations. Variables were hierarchically clustered based on similarity patterns. Strong positive correlations were observed between total flavonoid content (TFC) and both DPPH scavenging activity and α-amylase inhibition, while sugars exhibited predominantly negative correlations with several phenolic and flavonoid compounds. Statistical significance of correlations is indicated by asterisks: *p < 0.05; **p < 0.01; ***p < 0.001.

Pearson correlation analysis was performed to explore the relationships among key phytochemicals, antioxidant activity, and α-amylase inhibition (Fig. 5). Total flavonoid content (TFC) exhibited very strong positive correlations with DPPH scavenging activity (*r* = 0.97, *p* = 0.001) and α-amylase inhibition (*r* = 0.95, *p* = 0.004). In contrast, total phenolic content (TPC) showed relatively weak positive correlations with DPPH activity (*r* = 0.10, *p* > 0.05) and α-amylase inhibition (*r* = 0.12, *p* > 0.05). Among the identified phenolic acids, protocatechuic acid (*r* = 0.69 and *r* = 0.75) and ferulic acid (*r* = 0.68 and *r* = 0.74) exhibited comparatively strong positive associations with DPPH radical scavenging activity and α-amylase inhibition, respectively. Sinapic acid also exhibited relatively strong positive associations (*r* = 0.62 and *r* = 0.70), followed by 2,4-dihydroxybenzoic acid (*r* = 0.56 and *r* = 0.64) and caffeic acid (*r* = 0.56 and *r* = 0.66). In contrast, vanillic acid showed strong negative correlations with both DPPH activity (*r* =  − 0.89) and enzyme inhibition (*r* =  − 0.84).

Among the individual flavonoids, catechin showed strong positive correlations with both DPPH radical scavenging activity (*r* = 0.84, *p* = 0.035) and α-amylase inhibition (*r* = 0.76). Hesperetin also exhibited a strong positive correlation with DPPH activity (*r* = 0.83, *p* = 0.040) and a positive association with α-amylase inhibition (*r* = 0.75). Rutin (*r* = 0.56 and *r* = 0.66) and myricetin (*r* = 0.55 and *r* = 0.43) displayed comparatively moderate correlations. Total anthocyanin content (TAC) displayed moderate correlations with DPPH activity (*r* = 0.56) and α-amylase inhibition (*r* = 0.44), with its individual fractions delphinidin (*r* = 0.61 and *r* = 0.49) and cyanidin (*r* = 0.47 and *r* = 0.36) following a similar trend.

A very high positive correlation was observed between DPPH scavenging activity and α-amylase inhibition (*r* = 0.98, *p* < 0.001). Furthermore, IC_50_ values showed strong negative correlations with TFC (*r* =  − 0.83, *p* = 0.041), DPPH activity (*r* =  − 0.83, *p* = 0.042), and α-amylase inhibition (*r* =  − 0.87, *p* = 0.024), whereas a comparatively moderate negative correlation was observed with TPC (*r* =  − 0.46, *p* > 0.05).

#### KEGG pathway analysis of phytochemical biosynthesis in *Syzygium cumini*

**Fig. 6:**
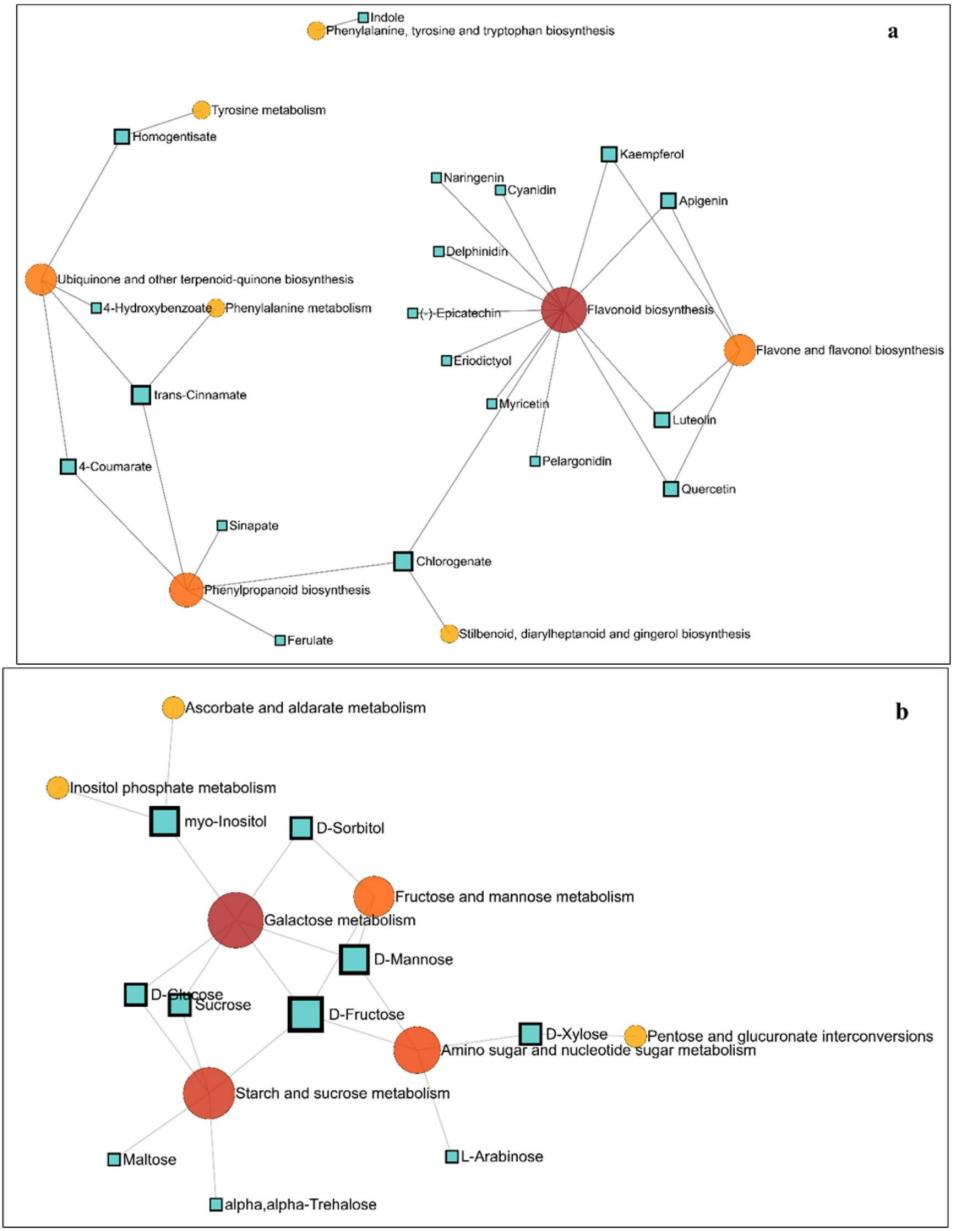
KEGG‑based metabolite pathway interaction networks of jamun. Metabolites detected in all three biological replicates (*n* = 3) across the six genotype–tissue combinations (3 genotypes × 2 tissues) were used for pathway mapping. (**a**) Secondary metabolite biosynthesis network. Flavonoid biosynthesis emerges as a central hub, connecting quercetin, kaempferol, luteolin, and epicatechin to the phenylpropanoid pathway via trans‑cinnamate and p-coumarate. (**b**) Carbohydrate metabolism network. Galactose metabolism functions as the core of the carbohydrate cluster, linking glucose, fructose, sucrose, and mannose to starch/sucrose and fructose/mannose metabolism. Circular nodes represent KEGG pathways, square nodes represent identified metabolites, edges indicate metabolite–pathway associations. The networks were adapted from the KEGG database (http://www.kegg.jp/kegg/kegg1.html) with permission from Kanehisa Laboratories. partitioning.

KEGG (Kyoto Encyclopedia of Genes and Genomes) pathway metabolite network analysis revealed that the detected metabolites in jamun pulp and seed samples were highly interconnected with secondary metabolite biosynthesis and carbohydrate metabolism (Fig. 6). The flavonoid biosynthetic pathway involves key metabolites, including quercetin, kaempferol, luteolin, and epicatechin. These compounds are biosynthetically linked to the phenylpropanoid pathway through intermediates such as trans-cinnamate and p-coumarate.

**Fig. 7:**
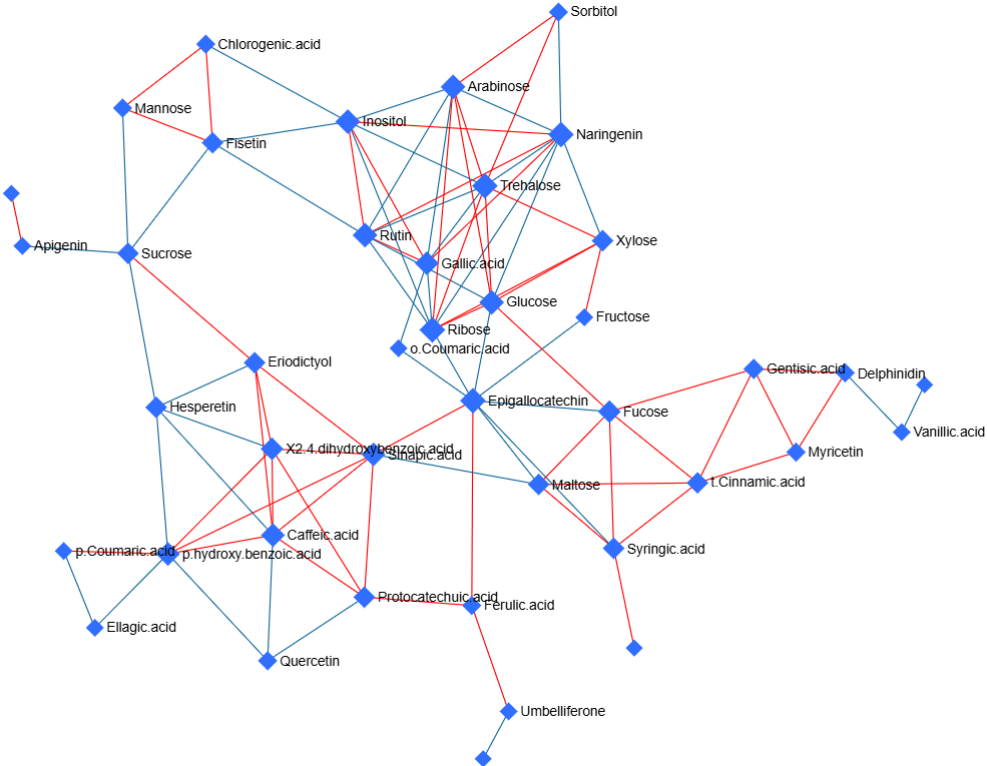
Network analysis of metabolite interactions in jamun *Syzygium cumini* (L.) Skeels genotypes. Correlation network displaying metabolite–metabolite associations (*r* > 0.70). Red edges represent positive correlations, while blue edges denote negative correlations. The network highlights strong clustering among phenolic acids and flavonoids forming the metabolic core, whereas sugars exhibit negative associations, reflecting contrasting primary and secondary metabolic regulation.

In addition to secondary metabolism, galactose metabolism represented a central carbohydrate-associated pathway, connecting major sugars including glucose, fructose, sucrose, and mannose with starch/sucrose and fructose/mannose metabolic pathways. Correlation network analysis further revealed that most sugars, including glucose, fructose, maltose, trehalose, and sucrose, formed a predominantly negative association cluster with phenolics and flavonoids (Fig. 7). Among these, fructose and sucrose exhibited the strongest negative correlations (*r* <  − 0.70) with metabolites such as caffeic acid, ferulic acid, and quercetin.

## Discussion

### Phenolic acid signatures and their functional implications

Genotype-specific variation in phenolic acids composition indicates strong metabolic regulation of secondary metabolite biosynthesis in pulp and seed of *Syzygium cumini*. The predominance of benzoic and gallic acids across the studied genotypes highlights the importance of phenylpropanoid metabolism in shaping jamun phytochemical composition. The enrichment of hydroxybenzoic and hydroxycinnamic acids resembles metabolic patterns reported in berry fruits, suggesting that conserved phenylpropanoid pathways contribute to antioxidant specialization across anthocyanin-rich fruit species^21,22^. Moreover, gallic acid, ellagic acid, and caffeic acid have been identified as major bioactive constituents in a polyherbal immunomodulatory formulation, reinforcing their broad functional relevance^23^. The elevated gallic acid content in PCJ-9 and PCJ-17 supports its recognized radical-scavenging activity^24^, while the high ellagic acid accumulation in PCJ-9 agrees with earlier reports identifying jamun seed ellagitannins as major contributors to antioxidant and antiproliferative potential^25,26^. Differential distribution of hydroxybenzoic and hydroxycinnamic acid derivatives further suggests distinct physiological roles related to sensory quality and oxidative stability^27^. The broader phenolic diversity observed in PCJ-15 and selective enrichment of gallic and ellagic acids in PCJ-9 indicate genotype-dependent phenolic biosynthesis and metabolite partitioning, consistent with previous findings in jamun and other underutilized fruits^28^.

### Flavonoid distribution and functional significance

Distinct genotype-dependent flavonoid profiles observed in *Syzygium cumini* indicate strong regulation of flavonoid biosynthesis among the studied genotypes. The predominance of myricetin, catechin, hesperetin, and rutin agrees with earlier reports describing jamun as a rich source of bioactive flavonoids^27,29^. Flavonoid accumulation in pulp tissues is closely associated with active phenylpropanoid metabolism, contributing to antioxidant defense, pigmentation, and fruit quality attributes^30^. In particular, myricetin and catechin are well recognized for their strong antioxidant and metal-chelating properties^31,32^, whereas hesperetin and rutin are associated with cardioprotective, anti-inflammatory, and vasoprotective activities^33,34^. The enrichment of rutin and quercetin highlights the functional specialization of jamun tissues toward accumulation of flavonols associated with antioxidant and antidiabetic activities^35–39^. Rutin has been widely associated with glucose regulation, α-glucosidase inhibition, and antihypertensive activity^37^, and quercetin, also identified as a prominent immunomodulatory metabolite in polyherbal preparations^23^, contributes significantly to antioxidant and antidiabetic potential^33^. The conserved flavonoid partitioning observed across jamun and other fruit crops suggests that tissue-specific allocation of antioxidant flavonoids represents a common metabolic strategy in fruit development^40–42^.

### Anthocyanin partitioning and functional implications

Pronounced genotype-dependent variation in anthocyanin composition indicates differential regulation of pigment biosynthesis *Syzygium cumini*. The predominance of cyanidin and delphinidin supports earlier reports identifying jamun as a rich source of antioxidant anthocyanins^29–32^. These pigments are widely associated with radical scavenging, cardioprotective, and anti-inflammatory activities^43–45^, while malvidin has additionally been linked with neuroprotective and anticancer potential^46^. The consistently low pelargonidin content suggests its limited contribution to jamun pigmentation. The limited contribution of pelargonidin suggests that jamun pigmentation is primarily governed by cyanidin- and delphinidin-derived pathways, similar to metabolic trends observed in other dark-colored berry fruits^47^. The predominance of cyanidin and delphinidin highlights the importance of anthocyanins in pigmentation and antioxidant functionality in jamun fruit tissues^26,39^. Such differential allocation of pigmented and non-pigmented phenolics is associated with distinct physiological functions related to pigmentation, antioxidative protection, and long-term defense metabolism^43,44^. The observed genotype and tissue interaction highlights the potential of jamun for functional food development and bioactive-oriented breeding programs^29–42^.

### Carbohydrate patterns and their functional significance

Distinct variation in sugar composition among the evaluated *Syzygium cumini* genotypes indicates substantial diversity in carbohydrate metabolism and metabolite allocation. The predominance of glucose and fructose reflects active sugar accumulation during ripening and likely contributes directly to fruit sweetness, consumer acceptability, and energy metabolism^48–51^. Variations in minor sugars such as arabinose, xylose, rhamnose, and fucose further support genotype-specific regulation of primary metabolism in jamun fruits^49^.

Seed tissues showed relatively greater enrichment of storage-associated sugars such as glucose and mannose. Such tissue-specific sugar partitioning is metabolically important because fructose-rich pulp contributes to sweetness and glycaemic response, while seed carbohydrates are more closely associated with metabolic stability and storage functions^48,49^. The predominance of low sucrose levels further supports the characterization of jamun as a comparatively low-glycaemic fruit, which may enhance its dietary suitability for glycaemic management^50,51^. In addition, the presence of minor sugars and seed-associated bioactives may synergistically contribute to the reported antidiabetic properties of jamun by modulating carbohydrate-hydrolysing enzymes and glucose metabolism.

### Multivariate metabolic discrimination and biomarker identification between pulp and seed

The distinct separation of pulp and seed tissues in OPLS-DA score plots highlights pronounced tissue-specific metabolic organization in *Syzygium cumini*, indicating that differential allocation of phenolic acids, flavonoids, anthocyanins, and sugars clearly shapes the phytochemical and functional characteristics of jamun. Similar tissue-dependent metabolic discrimination has also been reported in cashew apple^52^, pomegranate^53^, and other fruit metabolomics studies^54^, supporting the effectiveness of OPLS-DA for tissue-oriented phytochemical characterization. The reliability of such metabolomic findings is underpinned by validated UHPLC-MS/MS methods for secondary metabolite quantification, as recently demonstrated for bioactive oxygen heterocyclic compounds and terpenes in grapefruit essential oils^55^. The high R^2^ and Q^2^ values together with significant permutation testing further confirmed the robustness, predictive accuracy, and statistical reliability of the generated models, consistent with previous metabolomics studies in cashew apple^56^ and muskmelon^57^. Furthermore, VIP score analysis identified several phenolic acids, flavonoids, anthocyanins, and sugars as key discriminatory metabolites contributing to tissue differentiation. Many of these high-VIP metabolites are widely recognized for their antioxidant and health-promoting properties, suggesting their important contribution to the functional and nutraceutical potential of jamun. The identification of high-VIP metabolites highlights their central contribution to tissue-specific metabolic discrimination and functional specialization in *Syzygium cumini*. Similar metabolite-driven differentiation has been reported in citrus^58^, cashew apple^56^, and Gannan orange^59^, indicating that multivariate metabolomics approaches are highly effective for identifying bioactive biomarkers associated with phytochemical functionality and nutritional traits. Comparable UPLC–MS-based profiling strategies have also been successfully applied for tracking bioactive metabolites in medicinal plant systems^60^.

### Variation in bioactivities

Seed extracts exhibited stronger antioxidant activity than pulp extracts, consistent with previous reports in *Syzygium cumini*, where seeds accumulate higher levels of gallic acid, ellagic acid, catechins, and tannins^37,60^. Such genotype-dependent variation indicates substantial diversity in polyphenol biosynthesis among jamun landraces, directly influencing antioxidant efficiency^61^. These polyphenols possess multiple hydroxyl groups that enhance electron-transfer and proton donation reactions, thereby improving radical-scavenging efficiency and lowering IC_50_ values^62^. The superior DPPH activity observed in PCJ-15 may therefore be associated with greater accumulation of flavonoids and other antioxidant phenolics, particularly in seed tissues^63^. A similar tissue-dependent trend was observed for α-amylase inhibition, with seed extracts showing stronger inhibitory activity than pulp extracts. This response is likely linked to the higher abundance of phenolics, flavonoids, tannins, glycosides, and saponins in jamun seeds^61^. Phenolic compounds inhibit α-amylase through hydrogen bonding and hydrophobic interactions with catalytic residues of the enzyme^62^.

The strong α-amylase inhibitory activity observed in PCJ-15 suggests enhanced accumulation of bioactive polyphenols capable of suppressing pancreatic α-amylase activity and reducing starch hydrolysis and postprandial glucose release^64,65^. These findings highlight the nutraceutical potential of phytochemical-rich jamun genotypes as natural antioxidant and antidiabetic agents.

### Correlation of specific bioactivities with phytochemicals

To further identify which phytochemicals were primarily responsible for these activities, Pearson correlation analysis was performed. The strong positive association of total flavonoid content with DPPH scavenging and α‑amylase inhibition indicates that flavonoids are the principal contributors to the antioxidant and antidiabetic potential of *Syzygium cumini*. The strong association between flavonoids and bioactivities reinforces the central role of hydroxylated flavonoid structures in both radical scavenging and enzyme inhibition^66^. Protocatechuic, ferulic, sinapic, and caffeic acids also showed strong positive associations with bioactivity, consistent with reports emphasizing the importance of hydroxylated phenolic structures in free‑radical neutralization and enzyme inhibition^67–69^ whereas vanillic acid showed an inverse relationship.

Among flavonoids, catechin, hesperetin, rutin, and myricetin were strongly associated with antioxidant and α‑amylase inhibitory activities, supporting earlier reports describing flavan‑3‑ols and flavonols as effective radical scavengers and carbohydrate‑hydrolysing enzyme inhibitors^70,71^. Anthocyanins exhibited comparatively moderate associations, indicating a complementary rather than dominant contribution to bioactivity^72,73^. The strong correlation between antioxidant and α‑amylase inhibitory activities further suggests that common polyphenolic constituents contribute simultaneously to both functions. These findings highlight the central role of polyphenols, particularly flavonoids, in defining the functional and nutraceutical value of *Syzygium cumini*.

### Pathway-level interplay between phenolic biosynthesis and carbohydrate metabolism

The integration of detected metabolites into phenylpropanoid and flavonoid biosynthetic pathways confirms that a highly active secondary metabolite system underpins the medicinal and nutraceutical properties of *Syzygium cumin*^74,75^. Within the carbohydrate network, galactose metabolism centrally links major sugars to starch and sucrose metabolism. Pulp preferentially accumulates soluble sugars linked to ripening, whereas seed aligns with starch metabolism, consistent with a storage‑reservoir role, reflecting source–sink partitioning during fruit development^76,77^. Notably, primary sugars correlated negatively with phenolics and flavonoids, particularly fructose and sucrose with caffeic acid, ferulic acid, and quercetin, suggesting metabolic competition between carbon allocation toward primary carbohydrate metabolism and secondary metabolite biosynthesis during fruit development, similar to metabolic trade-offs reported in apple^78^. This differential metabolite allocation suggests that anthocyanins and sugar-rich pulp tissues may contribute more to fruit colour, palatability, and nutritional quality, whereas seeds represent a concentrated source of health-promoting phenolics and flavonoids with greater functional bioactivity.

Across all metabolite classes, a clear tissue-specific metabolic partitioning pattern was evident in *Syzygium cumini*. Seed tissues preferentially accumulated phenolic acids and flavonoids associated with antioxidant and α-amylase inhibitory activities, whereas pulp tissues were enriched with anthocyanins and soluble sugars contributing to pigmentation, palatability, and nutritional quality. This differential metabolite allocation reflects distinct physiological functions, with seeds functioning as reservoirs of defense-related phytochemicals and pulp supporting fruit attractiveness and energy metabolism. Consequently, the stronger bioactivities observed in seed extracts were closely associated with their greater polyphenol density, highlighting the potential of jamun seeds as valuable sources of nutraceutical bioactives and functional ingredients.

## Conclusions

This study provides a high-resolution metabolomic characterization of *Syzygium cumini* (L.) Skeels using integrated tissue-specific, genotype-specific, and pathway-level analyses. The results demonstrated that the bioactive potential of jamun is associated more strongly with specific flavonoid subclasses than with total phenolic concentration alone. A clear tissue-specific metabolite partitioning pattern was observed, with seeds accumulating higher levels of phenolic acids and flavonoids, while pulp tissues were enriched in anthocyanins and sugars. Consistent with this metabolic allocation, seed extracts exhibited superior antioxidant and α-amylase inhibitory activities, with catechin and protocatechuic acid identified as major contributors to bioactivity through correlation and multivariate analyses. The inverse relationship between sugar accumulation and secondary metabolite synthesis further highlighted genotype PCJ-15 as a promising low-glycemic and phytochemical-rich genetic resource. In addition, KEGG-based pathway mapping revealed important links between phenylpropanoid metabolism and functional bioactivity. Overall, these findings advance the understanding of metabolite–bioactivity relationships in jamun and provide a valuable foundation for future nutraceutical development, functional food applications, and genotype improvement programs.

## Materials and methods

### Plant material and genotype selection

Three contrasting *Syzygium cumini* (jamun) genotypes (PCJ-9, PCJ-17, and PCJ-15) were selected from a large seedling-origin population maintained at the ICAR-Indian Agricultural Research Institute (IARI), New Delhi (28.40° N, 77.10° E), based on our previously characterized morpho-biochemical and bioactive diversity^79^. PCJ-9 and PCJ-17 are large-fruited genotypes exhibiting high pulp weight, pulp-to-seed ratio, total soluble solids, and vitamin C, but relatively low total phenolic content. In contrast, PCJ-15 is a small-fruited genotype distinguished by a markedly higher seed proportion, elevated total phenolic and flavonoid content, and superior antioxidant activity. These contrasting characteristics made the selected genotypes suitable for comparative metabolomic and bioactivity analyses. Fully ripened fruits were collected from healthy trees of uniform age and growth conditions at the same experimental site. Fruits were washed, manually separated into pulp and seed, immediately frozen in liquid nitrogen, and stored at − 80 °C until metabolite extraction and LC–MS/MS analysis.

### Determination of phenolic acids and flavonoids by LC–MS/MS

#### Extraction procedure

Phenolic acids and flavonoids were extracted by an altered procedure^80,81^. In short, 80% methanol was used to extract freeze-dried powdered sample, then sonication for 20 min was applied and the centrifugation at 5,000 × g for 10 min at 4 °C was performed; the residue was extracted two more times and the pooled supernatants were evaporated under vacuum at a temperature of 45 °C and then dissolved again in 5 mL of acidified aqueous methanol (0.1% formic acid) to get the free phenolic fraction. The solution was twice washed with n-hexane in order to waste the lipophilic impurities, and the aqueous phase underwent a filter (0.22 µm PTFE) for LC–MS/MS analysis. To find out bound phenolics, the residue that was left after methanolic extraction was hydrolyzed with 2 mL of 2 M NaOH under nitrogen for 2 h at room temperature, acidified to pH 2.0 with 6 M HCl, and then ethyl acetate was used to extract three times; the ethyl acetate phase that was separated was evaporated (≤ 40 °C), and the residue was dissolved in 1 mL of LC–MS-grade methanol and filtered before injection. Free (non-hydrolyzed) extracts were taken for the quantification of native flavonoid glycosides, while alkaline hydrolysates were utilized solely for bound phenolics.

#### LC–MS/MS conditions

Chromatographic separation was performed on an ACQUITY UPLC BEH C18 column (2.1 × 50 mm, 1.7 µm; Waters, USA) equipped with a Vanguard pre-column. The mobile phase consisted of (A) water with 0.1% formic acid and (B) acetonitrile with 0.1% formic acid. A linear gradient was applied at a flow rate of 0.1 mL min^−1^, and the column temperature was maintained at 25 °C. The injection volume was 6 µL. Online detection was achieved using a photodiode array (PDA) detector coupled to a TQD triple-quadrupole mass spectrometer (Waters, USA) equipped with an electrospray ionization source (ESI). Quantification was performed in multiple reaction monitoring (MRM) mode using optimized precursor–product ion transitions for each analyte.

### Anthocyanin analysis by LC–MS/MS

#### Extraction procedure

The extraction of anthocyanins was done according to a modified protocol^82^. In short, a sample of 1.5 g was finely homogenized and mixed with 20 mL of methanol containing 1% (v/v) HCl and incubated in the dark at 4 °C for 12 h with shaking at intervals. The extract was spun down (10,000 × g, 10 min, 4 °C) and the supernatant was taken. A portion of 1 mL from the liquid on top was evaporated to a dry state under reduced pressure at a temperature of 30 °C or less and was dissolved in 1 mL of the initial LC mobile phase. Before LC–MS/MS analysis, the sample was filtered using a 0.22 µm PTFE syringe filter to eliminate particulates.

#### LC–MS/MS conditions

The separation of anthocyanins using chromatography was carried out on an ACQUITY UPLC BEH C18 column (2.1 × 50 mm, 1.7 μm; Waters, USA) outfitted with a Vanguard pre-column. The solvents were (A) water with 0.25% formic acid and (B) acetonitrile with 0.25% formic acid. The elution was carried out by using a linear gradient and a flow rate of 0.25 mL min^−1^. The column was heated to 35 °C, and the volume for injection was 6 μL. The ACQUITY TQD triple quadrupole mass spectrometer (Waters, USA) was used for the detection and quantification of the anthocyanins.

### Sugar analysis by LC–MS/MS

#### Extraction procedure

Sugar extraction was performed following a modified method of Steppuhn and Wäckers^83^. Briefly, powdered samples were extracted twice with warm 80% ethanol under vortex mixing. The pooled extracts were centrifuged (10,000 × g, 10 min), and the supernatant was adjusted to 20 mL with 80% ethanol. An aliquot (1 mL) was evaporated under reduced pressure at ≤ 40 °C and reconstituted in ultrapure water containing 0.1% formic acid. After sonication and filtration through a 0.22 μm nylon membrane, the extract was diluted with the mobile phase and used for LC–MS/MS analysis.

#### LC–MS/MS conditions

The chromatographic separation of the soluble sugars was implemented on an Acquity UPLC BEH Amide column having a dimension of 2.1 × 100 mm with 1.7 μm particle size (Waters, USA) and equipped with a BEH Amide Vanguard pre-column. The mobile phases comprised: (A) acetonitrile and (B) 10 mM ammonium acetate solution in water (pH 4.5), both of which were filtered over (0.22 μm). A linear HILIC gradient was applied at a flow rate of 0.30 mL min^−1^: 90% A (0–1 min), 90–70% A (1–10 min), 70–50% A (10–13 min), 50–90% A (13–15 min), followed by re-equilibration at 90% A (15–18 min). The temperature of the column was held at 35 °C, and the volume of the sample to be injected was 2 μL. The detection was enabled using a photodiode array (PDA) detector that was coupled in-line to a TQD triple-quadrupole mass spectrometer (Waters, USA), which operated under multiple reaction monitoring (MRM) mode with the use of electrospray ionization.

#### Instrumentation and standards

All chromatographic analyses were performed on a Waters ACQUITY UPLC-H Class system (Waters, USA) connected to a TQD triple-quadrupole tandem mass spectrometer with an electrospray ionization (ESI) source. Only LC–MS-grade solvents were used for both sample preparation and mobile phases. All the standards (≥ 98% purity) were sourced from Sigma-Aldrich (USA), and the quantitative analysis was done with the help of external calibration curves made from the corresponding standards. Metabolite identification and quantification were achieved using optimized MRM transitions, retention time matching, and corresponding reference standards under identical chromatographic conditions. Mass spectrometric detection was performed in negative electrospray ionization (ESI–) mode, monitoring the predominant deprotonated molecular ions [M–H]^−^. These precursor ions were subjected to compound-specific collision energies to generate characteristic product ions, which were quantified using Multiple Reaction Monitoring (MRM). Detailed MRM parameters and representative chromatograms of sample extracts are provided in the supplementary information (Tables S1–S4; Figs. S1–S8). All LC–MS/MS analyses were performed using three independent biological replicates (*n* = 3) for each genotype and tissue type, and quantitative data are presented as mean ± SD.

### DPPH radical scavenging activity

The antioxidant capacity of the extracts was assessed using the DPPH (2,2-diphenyl-1-picrylhydrazyl) radical-scavenging assay, following the procedure^84^, with modifications as applied in earlier work on jamun biochemical profiling^79^. A DPPH stock solution was prepared by dissolving 25 mg of DPPH in 100 mL of methanol and adjusting the absorbance to 0.70 ± 0.01 at 517 nm. For sample extraction, 2 g of jamun fruit tissue was homogenized in 10 mL methanol and centrifuged at 10,000 × g for 20 min. An aliquot of 100 µL of the resulting supernatant was combined with 3.9 mL of the DPPH working solution and incubated in the dark for 2 h at room temperature. The decrease in absorbance at 517 nm was recorded against a reagent blank, and the percentage of DPPH radical scavenging was calculated by comparing the absorbance of the reaction mixture with the control.

$${\mathrm{DPPH}}\;{\mathrm{inhibition}}\left( {{\% }} \right) = \left\{ {\frac{{{\mathrm{Ac}} - {\mathrm{As}}}}{{{\mathrm{Ac}}}}} \right\} \times 100$$

where Ac = Absorbance (optical density) of DPPH; As = Absorbance of sample.

### α-amylase inhibitory activity

The α-amylase inhibitory activity of fruit extracts was evaluated using porcine pancreatic α-amylase (PPA; EC 3.2.1.1) following the 3,5-dinitrosalicylic acid (DNSA) colorimetric method described by Miller^85^ and as adopted in jamun antidiabetic studies by Gajera et al.^61^. Briefly, 200 µL of extract (0.1–300 µg/mL for IC_50_ determination) was pre-incubated with 200 µL of PPA solution (1 U/mL in 0.02 M phosphate buffer, pH 6.9, containing 0.006 M NaCl) at 37 °C for 10 min. The reaction was initiated by adding 200 µL of 1% (w/v) soluble starch, followed by incubation for 15 min at 37 °C. The reaction was terminated with 200 µL DNSA reagent, and the mixture was heated at 100 °C for 5 min, cooled, diluted with 2 mL distilled water, and the absorbance was measured at 540 nm. Enzyme control (without extract), sample color control, and reagent blank were included, while acarbose served as the positive standard. Percent inhibition was calculated after blank correction. The IC_50_ value, defined as the extract concentration required to inhibit 50% of PPA activity, was estimated from nonlinear regression of inhibition (%) versus log concentration. One unit of enzyme activity corresponded to the amount of enzyme releasing 1 µmol of maltose per minute under assay conditions.

### Statistical analysis

A two-way analysis of variance (ANOVA) was performed on quantitative LC–MS/MS and bioactivity datasets to evaluate the effects of genotype, tissue type, and their interaction. Mean comparisons were performed using Tukey’s honestly significant difference (HSD) post-hoc test at *p* < 0.05. The results are presented as mean ± standard deviation (SD) based on three independent biological replicates (*n* = 3). The RAISINS statistical environment^86^ was used for significance testing and graphical visualization. Multivariate analyses such as hierarchical clustering analysis (HCA), principal component analysis (PCA), correlation network analysis, and orthogonal partial least squares-discriminant analysis (OPLS-DA), KEGG pathway analysis were performed using MetaboAnalyst 6.0 (https://www.metaboanalyst.ca)^87^ and the Metware Biotechnology (MetwareBio) analytical platform (https://www.metwarebio.com). Identified metabolites were mapped to metabolic pathways using the Kyoto Encyclopedia of Genes and Genomes (KEGG) database (http://www.kegg.jp/kegg/kegg1.html)^88–90^. Formal permission for the use and adaptation of KEGG pathway imagery was obtained from Kanehisa Laboratories, and the results were used to visualize metabolite-pathway interaction networks.

## Supplementary Information

Below is the link to the electronic supplementary material.

Supplementary Material 1

## Data Availability

Data will be made available on request.
