# Supplementary material for: LC–MS/MS-based metabolomic profiling of Syzygium cumini (L.) Skeels identifies key phytochemicals associated with α-amylase inhibition and antioxidant activity
Source: Sci Rep. 2026 May 26;16:24107. doi: 10.1038/s41598-026-55512-x (PMC13438540; doi:10.1038/s41598-026-55512-x)
Supplement: Supplementary file 1 [file 41598_2026_55512_MOESM1_ESM.docx]

**Fig. S1**. Representative MRM chromatograms of phenolic acids in pulp samples of *Syzygium cumini* genotypes.

**Fig. S2.** Representative MRM chromatograms of phenolic acids in seed samples of *Syzygium cumini* genotypes.

**Fig. S3.** Representative MRM chromatograms of flavonoids in pulp samples of *Syzygium cumini* genotypes.

**Fig. S4**. Representative MRM chromatograms of flavonoids in seed samples of *Syzygium cumini* genotypes.

**Fig. S5.** Representative MRM chromatograms of anthocyanins in pulp samples of *Syzygium cumini* genotypes.

**Fig. S6.** Representative MRM chromatograms of anthocyanins in seed samples of *Syzygium cumini* genotypes.

**Fig. S7.** Representative MRM chromatograms of sugars in pulp samples of *Syzygium cumini* genotypes.

**Fig. S8.** Representative MRM chromatograms of sugars in seed samples of *Syzygium cumini* genotypes.

**Table S1.** MRM parameters of phenolic acid standards used for LC–MS/MS analysis.

| **Sl. No.** | **Compounds** | **Formula/Mass** | **Parent**  **ion (m/z) [M+H]^+^** | **Daughters** | **Cone**  **voltage**  **(V)** | **Collision**  **energy**  **(eV)** | **Ion**  **mode** |
| --- | --- | --- | --- | --- | --- | --- | --- |
| 1 | Caffeic acid | 180 | 178.90 | 135.05 | 30 | 16 | ES- |
| 2 | 2,4- Dihydroxybenzoic acid | 154 | 152.90 | 65.02 | 28 | 18 | ES- |
| 3 | Chlorogenic acid | 354 | 352.97 | 191.10 | 22 | 18 | ES- |
| 4 | Ferulic acid | 194 | 192.90 | 134.02 | 26 | 14 | ES- |
| 5 | Gallic acid | 170 | 168.90 | 125.03 | 28 | 12 | ES- |
| 6 | Gentisic Acid | 154 | 152.90 | 108.98 | 24 | 12 | ES- |
| 7 | *o*-Coumaric acid | 164 | 162.90 | 119.06 | 22 | 12 | ES- |
| 8 | *p*-Coumaric acid | 164 | 162.90 | 119.05 | 24 | 14 | ES- |
| 9 | *p*-Hydroxybenzoic acid | 138 | 136.90 | 93.01 | 26 | 12 | ES- |
| 10 | Protocatecuic acid | 154 | 152.90 | 109.05 | 26 | 16 | ES- |
| 11 | Salicylic acid | 138 | 136.90 | 93.10 | 28 | 14 | ES- |
| 12 | Syringic acid | 198 | 196.97 | 182.07 | 26 | 10 | ES- |
| 13 | t-Cinnamic Acid | 148 | 146.90 | 103.05 | 26 | 10 | ES- |
| 14 | Vanillic Acid | 168 | 166.97 | 108.01 | 26 | 20 | ES- |
| 15 | Benzoic acid | 122 | 120.96 | 77.02 | 26 | 10 | ES- |
| 16 | 3-Hydroxy Benzoic acid | 138 | 137.02 | 93.8 | 28 | 10 | ES- |
| 17 | Sinapic acid | 224 | 223.11 | 149.06 | 32 | 14 | ES- |
| 18 | Ellagic acid | 302 | 301.09 | 144 | 70 | 38 | ES- |

**Table S2.** MRM parameters of flavonoids standards used for LC–MS/MS analysis.

| **Sl.**  **No.** | **Compounds** | **Formula/Mass** | **Parent ion**  **(m/z) [M+H]^+^** | **Daughters** | **Cone** **voltage**  **(V)** | **Collision**  **energy**  **(eV)** | **Ion**  **mode** |
| --- | --- | --- | --- | --- | --- | --- | --- |
| 1 | Apigenin | 270 | 268.97 | 107.04 | 46 | 30 | ES- |
| 2 | Catechin | 290 | 289.03 | 245.15 | 38 | 12 | ES- |
| 3 | Hesperetin | 302 | 300.97 | 286.15 | 42 | 16 | ES- |
| 4 | Myricetin | 318 | 317.03 | 151.06 | 42 | 28 | ES- |
| 5 | Quercetin | 302 | 301.03 | 151.12 | 36 | 20 | ES- |
| 6 | Rutin | 610 | 609.10 | 300.20 | 60 | 42 | ES- |
| 7 | Umbelliferone | 162 | 161.04 | 133.07 | 42 | 18 | ES- |
| 8 | Luteolin | 286 | 284.97 | 93.05 | 52 | 32 | ES- |
| 9 | Kaemepherol | 286 | 284.97 | 93.05 | 52 | 32 | ES- |
| 10 | Naringenin | 272 | 271.13 | 119.05 | 34 | 22 | ES- |
| 11 | Epicatechin | 290 | 289.05 | 109.08 | 36 | 22 | ES- |
| 12 | Epigallocatechin | 306 | 305.05 | 125.10 | 34 | 20 | ES- |
| 13 | Fisetin | 286 | 285.08 | 121.05 | 42 | 26 | ES- |
| 14 | Galangin | 270 | 269.08 | 168.53 | 52 | 28 | ES- |
| 15 | Eriodictyol | 288 | 287.09 | 135.12 | 32 | 20 | ES- |

**Table S3.** MRM parameters of anthocyanins standards used for LC–MS/MS analysis.

| **Sl. No.** | **Compounds** | **Formula/Mass** | **Parent ion**  **(m/z) [M+H]^+^** | **Daughters** | **Cone voltage (V)** | **Collision energy (eV)** | **Ion**  **mode** |
| --- | --- | --- | --- | --- | --- | --- | --- |
| 1 | Cyanidin | 286.00 | 287.10 | 137.07 | 56 | 34 | ES+ |
| 2 | Delphinidin | 301.98 | 303.08 | 229.06 | 66 | 32 | ES+ |
| 3 | Malvidin | 330.00 | 331.16 | 315.11 | 40 | 34 | ES+ |
| 4 | Pelargonidin | 270.00 | 271.10 | 121.05 | 56 | 34 | ES+ |

**Table S4.** MRM parameters of sugar standards used for LC–MS/MS analysis.

| **Sl.**  **No.** | **Compounds** | **Formula/Mass** | **Parent ion**  **(m/z) [M+H]^+^** | **Daughters** | **Cone**  **voltage**  **(V)** | **Collision**  **energy**  **(eV)** | **Ion**  **mode** |
| --- | --- | --- | --- | --- | --- | --- | --- |
| 1 | Fructose | 180 | 178.97 | 89.09 | 18 | 16 | ES- |
| 2 | Sucrose | 342 | 341.03 | 89 .08 | 32 | 22 | ES- |
| 3 | Galactose | 180 | 178.97 | 89.09 | 18 | 16 | ES- |
| 4 | Glucose | 180 | 178.97 | 59.08 | 18 | 16 | ES- |
| 5 | Maltose | 342 | 341.03 | 161.05 | 12 | 8 | ES- |
| 6 | Fucose | 164 | 162.97 | 89.06 | 18 | 6 | ES- |
| 7 | Rhamnose | 164 | 162.97 | 103.06 | 18 | 6 | ES- |
| 8 | Xylose | 150 | 148.9 | 89.09 | 18 | 8 | ES- |
| 9 | Arabinose | 150 | 148.9 | 89.08 | 18 | 8 | ES- |
| 10 | Mannose | 180 | 178.97 | 119.03 | 20 | 8 | ES- |
| 11 | Sorbitol | 182 | 180.97 | 89.05 | 26 | 14 | ES- |
| 12 | Inositol | 180 | 178.97 | 161.11 | 28 | 8 | ES- |
| 13 | Lactose | 342 | 341.03 | 161.05 | 12 | 8 | ES- |
| 14 | Ribose | 150 | 148.9 | 89.09 | 18 | 8 | ES- |
| 15 | Trehalose | 342 | 341.03 | 89.08 | 32 | 22 | ES- |
